# Maternal Rat Diabetes Mellitus Deleteriously Affects Insulin Sensitivity and Beta-Cell Function in the Offspring

**DOI:** 10.1155/2013/429154

**Published:** 2013-08-12

**Authors:** Abdel-Baset M. Aref, Osama M. Ahmed, Lobna A. Ali, Margit Semmler

**Affiliations:** ^1^Cell Biology and Histochemistry Division, Zoology Department, Faculty of Science, South Valley University, Qena, Egypt; ^2^Physiology Division, Zoology Department, Faculty of Science, Beni-Suef University, Salah Salem Street, P.O. Box 62514, Beni-Suef, Egypt; ^3^Faculty of Oral and Dental Medicine, Nahda University, New Beni-Suef City, Beni-Suef, Egypt; ^4^Diabetes Research Institute, Düsseldorf, Germany

## Abstract

This study was designed to assess the effect of maternal diabetes in rats on serum glucose and insulin concentrations, insulin resistance, histological architecture of pancreas and glycogen content in liver of offspring. The pregnant rat females were allocated into two main groups: normal control group and streptozotocin-induced diabetic group. After birth, the surviving offspring were subjected to biochemical and histological examination immediately after delivery and at the end of the 1st and 2nd postnatal weeks. In comparison with the offspring of normal control dams, the fasting serum glucose level of offspring of diabetic mothers was significantly increased at the end of the 1st and 2nd postnatal weeks. Serum insulin level of offspring of diabetic dams was significantly higher at birth and decreased significantly during the following 2 postnatal weeks, while in normal rat offspring, it was significantly increased with progress of time. HOMA Insulin Resistance (HOMA-IR) was significantly increased in the offspring of diabetic dams at birth and after 1 week than in normal rat offspring, while HOMA insulin sensitivity (HOMA-IS) was significantly decreased. HOMA beta-cell function was significantly decreased at all-time intervals in offspring of diabetic dams. At birth, islets of Langerhans as well as beta cells in offspring of diabetic dams were hypertrophied. The cells constituting islets seemed to have a high division rate. However, beta-cells were degenerated during the following 2 post-natal weeks and smaller insulin secreting cells predominated. Vacuolation and necrosis of the islets of Langerhans were also observed throughout the experimental period. The carbohydrate content in liver of offspring of diabetic dams was at all-time intervals lower than that in control. The granule distribution was more random. Overall, the preexisting maternal diabetes leads to glucose intolerance, insulin resistance, and impaired insulin sensitivity and **β**-cell function in the offspring at different postnatal periods.

## 1. Introduction

Maternal health plays a significant role in determining as well as predicating the health of the offspring later in their life [1]. Fetal exposure to maternal diabetes *in utero* increases the risk of obesity/adiposity, glucose intolerance, and type 2 diabetes for offspring in later life [2–5]. Little information is available to explain the mechanism of these actions.

Animal studies revealed that the offspring of diabetic rats have been shown to be insulin resistant [6, 7] and diabetic [6, 8]. In the case of severe maternal diabetes in the spontaneously diabetic BB rat, this effect has also been associated with a reduction in the pancreatic insulin-positive cell mass [9]. Song et al. [10], Xu and Han [11], and Chavey et al. [5] demonstrated that maternal diabetes induces many metabolic and functional aberrations in adult offspring pancreatic islets that lead to impaired insulin secretion. They suggested that these aberrations may contribute to the development of NIDDM in later life of the offspring of diabetic mothers.

A variety of diabetic animal models during pregnancy are used to assess long-term effects on the offspring. A concern of studies using STZ during pregnancy is the possibility that the toxin might cross the placenta and be directly harmful to the fetal pancreas and other fetal tissues, thus, making any analysis of the long term effects of hyperglycemia *in utero* difficult [12]. Thus, the preexisting streptozotocin-induced diabetes mellitus in pregnant rats was most commonly used by several authors [13].

In conduction with the previous studies, the current study aims to investigate the effect of preexisting experimentally induced diabetes mellitus in rat dams on the glycemic status, insulin resistance, and *β*-cell function and integrity in their offspring at different postnatal periods.

## 2. Materials and Methods

### 2.1. Experimental Animals

Experiments were carried out on 65 white albino rats (*Rattus norvegicus*), 55 mature virgin females weighing about 170–200 g, and 10 mature males 190–220 g. The animals were obtained from the Animal House, Faculty of Medicine, Assiut University, Egypt. All animal procedures are in accordance with the general guidelines of animal care and the recommendations of the Canadian Committee of Canadian Council on Animal Care [14]. All efforts were made to minimize the number of animals used and their suffering.

Adult rats were kept under observation for 2 weeks before experimentation to exclude any intercurrent infection and to acclimatize the animals to the new conditions. The selected animals were marked, housed in stainless steel cages with separate bottom, and kept at a temperature of 23 ± 2°C, with good ventilation and a relative humidity of 50 ± 5%. The animals were exposed to constant light/dark periods of 12 hours (hr) each (light on at 06:00 hr) and fed on standard rodent pellet diet as well as some vegetables as a source of vitamins. For drinking tap, water was provided *ad libitum*.

### 2.2. Induction of Diabetes Mellitus

Diabetes mellitus was experimentally induced in female virgin animals fasted for 16 hours by intraperitoneal injection of 45 mg/kg b.wt. streptozotocin (Sigma-Aldrich Chemie GmbH, Germany) dissolved in citrate buffer (pH 4.5) [15, 16]. Ten days after streptozocin injection, rats were deprived of food and water overnight and blood samples were obtained from lateral tail vein after two hours of oral glucose loading (3 g/kg b.w.) Serum glucose level was measured for each female rat. Rats with serum glucose level higher than 180 mg/dL were considered as diabetic and were included in the experiment, while others were excluded.

### 2.3. Mating and Fertilization

To determine the estrus cycle, the vaginal smear of each virgin female was examined daily. Three types of cells, leukocytes and epithelial and cornified cells, were observed in photomicrographs of unstained vaginal smear. As reported by Marcondes et al. [17], the proportion of the three types of cells was used for the determination of the estrous cycle phases. A proestrus smear consists of a predominance of nucleated epithelial cells; an estrous smear primarily consists of anucleated cornified cells; a metestrus smear consists of the same proportion among leukocytes, cornified, and nucleated epithelial cells; a diestrus smear primarily consists of a predominance of leukocytes.

Proesterous normal and diabetic females were left for one night to copulate with the normal males (2 females with one male). Early next morning (before 7 am), copulation was checked by examining the outer surface of the vagina for the presence of a vaginal plug formed by coagulation of semen (white clotting, sperm clot). When such a grayish-white clot blocking the mouth of vagina was detected, this day was considered as the first day of gestation.

### 2.4. Pregnancy and Delivery

Each pregnant female was transferred into a separate cage and the weight gain was followed up throughout the pregnancy. Total numbers of 9 normal pregnant rats and 46 diabetic pregnant rats were included in the experiments.

After the birth, by the end of the 1st and 2nd postnatal weeks, blood and tissue samples of offspring of normal control and diabetic female dams were taken.

### 2.5. Experimental Investigation

#### 2.5.1. Biochemical Investigation

Blood samples of overnight fasted offspring of both groups were taken from jugular vein and centrifuged. The obtained sera from offspring were pooled within each litter and kept at −30°C until use.

#### 2.5.2. Determination of Serum Glucose Concentration

The obtained serum of offspring of normal and diabetic dams was used for determination of glucose concentration according to the method of Trinder [18], using reagent kits obtained from Human GmbH, Wiesbaden, Germany.

#### 2.5.3. Determination of Insulin Concentration

Serum insulin concentration of offspring of normal and diabetic dams was determined with radioimmunoassay kits of DPC (Diagnostic Products Corporation, Los Angeles, CA, USA) (Coat-A-Count) according to the method of Marschner et al. [19].

#### 2.5.4. Homeostatic Model Assessment (HOMA)

Insulin resistance (HOMA-IR), insulin sensitivity (HOMA-IS), and beta-cell function (HOMA-*β* cell function) were calculated according to Hsing et al. [20] and Park et al. [21] as follows.

*HOMA-IR. *This value is calculated according to the following equation:

(1) $$\begin{array}{c} \text{HOMA-IR}=\frac{\text{fasting}\;\text{insulin}\times \text{fasting}\;\text{glucose}}{405}. \end{array}$$

*HOMA-IS. *This value is calculated according to the following equation:

(2) $$\begin{array}{c} \text{HOMA-IS}=\frac{10000}{\text{fasting}\;\text{insulin}\times \text{fasting}\;\text{glucose}}. \end{array}$$

*HOMA-*β* Cell Function*. The beta-cell function is calculated according to the following equation:

(3) $$\begin{array}{c} \text{HOMA-}\beta \;\text{cell}\;\text{function}=\frac{20\times \text{fasting}\;\text{insulin}}{\text{fasting}\;\text{glucose}-3.5}. \end{array}$$

### 2.6. Microscopic Examination of Specific Organs

At specific time intervals (zero time, after one and 2 weeks), offspring of both normal and diabetic dams were sacrificed and liver and pancreas were immediately excised. Small tissue blocks were fixed in 10% neutral buffered formalin, embedded in paraffin wax, and cut serially at 5 *µ*m thickness. Pancreas sections were stained with modified aldehyde fuchsin stain according to Bancroft and Stevens [22] to discriminate the cell types of the islets of Langerhans. Liver sections were stained with Periodic Acid Schiff (PAS) reagent. Micrographs were taken using 40x light microscope.

### 2.7. Statistical Analysis

The data are analyzed by one-way analysis of variance (ANOVA) using PC-STAT, University of Georgia, followed by LSD analysis to discern the main effects and to compare various groups with each other [23]. *F*-probability for each variable expresses the general effect between groups. A two-way analysis of variance was also applied to evaluate the effect of time, diabetes, and their interaction during the experimental periods. The data are presented as mean ± standard error (SE) and values of *P* > 0.05 are considered statistically nonsignificant, while those of *P* < 0.05, *P* < 0.01, and *P* < 0.001 are considered statistically significant, highly significant, and very highly significant, respectively.

## 3. Results

To investigate the effect of preexisting maternal diabetes on development of newborn, offspring of normal control and diabetic females were examined at birth, after one and two weeks after delivery.

### 3.1. Biochemical Features of Offspring

#### 3.1.1. Serum Glucose (Table 1)

At birth, the serum glucose of normal control offspring showed a mean of 64.4 ± 7.8 mg/dL, increased significantly by 52.6% to 98.3 ± 8.3 mg/dL after 1 week, and remained constant until the end of the experimental period.

The serum glucose concentration of diabetic dam offspring was at birth 81.8 ± 8.8 mg/dL which was 27% higher than that of normal control, but insignificantly different. During the next two weeks, the glucose level was further increased to 121.7 ± 6.2 mg/dL and 131.9 ± 3.9 mg/dL, respectively. At theses time intervals, the mean values were significantly higher at 1% level higher than that of control neonates.

The increases in glucose levels of diabetic dam offspring were 39.9 mg/dL (+48.8%) from birth to 1st week and 10.2 mg/dL (+8.4%) from 1st to 2nd week.

Two-way ANOVA revealed that while the effect of time or diabetes is very highly significant (*P* < 0.001), the effect of their interaction is nonsignificant (*P* > 0.05).

#### 3.1.2. Insulin Concentration (Table 2)

At birth, the insulin concentration of normal control dam offspring showed a mean of 1.75 ± 0 *µ*U/mL. The hormone level was increased to 2.9 ± 0.001 *µ*U/mL and to 3.43 ± 0.09 *µ*U/mL after the first week and second postnatal week, respectively, revealing significant increases with time of 1.2 *µ*U/mL (+65.7%) from birth to 1st week and of 0.5 *µ*U/mL (+18.3%) during the 2nd postnatal week.

The insulin concentration of diabetic group offspring was at all-time intervals significantly different at a 1.0% level from that of control neonates. At birth, the hormone level was 2.4 ± 0.06 *µ*U/mL significantly higher (+37.1%) compared to normal control, while after the following time intervals, the insulin concentration was significantly decreased to 1.75 ± 0.02 *µ*U/mL, and 1.55 ± 0.02 *µ*U/mL respectively, being −39.7% and 54.8% lower than that of normal control neonates. The insulin level of diabetic dam offspring was reduced by −0.7 *µ*U/mL (−27.1%) and −0.2 *µ*U/mL (−11.4%) from birth to 1st week and from 1st week to 2nd week, respectively.

The analysis of the two-way ANOVA revealed that effect of time, diabetes, and their interaction is very highly significant throughout the experiment.

#### 3.1.3. Homeostatic Model Assessment (HOMA)

This model was used to estimate insulin resistance (IR), insulin sensitivity (IS), and the *β*-cell function.

*HOMA-IR (Table 3).* The calculated HOMA-IR value of normal control offspring showed means of 0.29 ± 0.02 at birth, of 0.46 ± 0.02 after one week, and of 0.54 ± 0.05 by the end of the experimental period.

The HOMA-IR of diabetic dam offspring was at birth and after one week significantly higher than that of control, while after the second week, the mean of both groups differed insignificantly. The differences between groups were +162%, +36.9%, and +11.1%, respectively. The decreases of HOMA-IR of diabetic dam offspring were −0.13 (−17.1%) and −0.03 (−4.8%) from birth to 1st week, and from 1st week to 2nd week, respectively.

The analysis of the two-way ANOVA showed that while the effect of diabetes or its interaction with time was very highly significant (*P* < 0.001), the effect of time alone is only significant.

*HOMA-IS (Table 4)*. At birth, the insulin sensitivity of normal dam offspring showed a mean of 151.3 ± 11.8. After one week, the value was significantly reduced by 64.1% to 54.2 ± 3.1. After two weeks, the sensitivity decreased insignificantly further to a mean of 47.2 ± 4.3.

The insulin sensitivity of diabetic dam offspring was at birth significantly lower (−68.9%) than that of control showing a mean of 47.1 ± 1.9. From birth to the end of the experimental period, the mean values decreased slightly further and remained until the end of the experimental period within the same range.

The analysis of the two-way ANOVA showed that the effect of time, diabetes, and their interaction is very highly significant throughout the experiment.

*HOMA-*β* Cell Function (Table 5)*. At birth, calculated beta-cell function of normal offspring showed a mean of 1.03 ± 0.08. During the following two weeks, the values varied insignificantly showing that the beta-cell function remained constant throughout the experimental period.

The calculated beta-cell function of diabetic dam offspring was at all-time intervals significantly lower than that of control. Furthermore, the values decreased with time showing, by the end of the experiment, a difference of −81.3% compared to control.

The analysis of the two-way ANOVA showed that the effect of time, diabetes, and their interaction is significant (*P* < 0.05), very highly significant (*P* < 0.001), and highly significant (*P* < 0.01), respectively.

### 3.2. Microscopic Features of Offspring

#### 3.2.1. Pancreas

**Figure 1:**
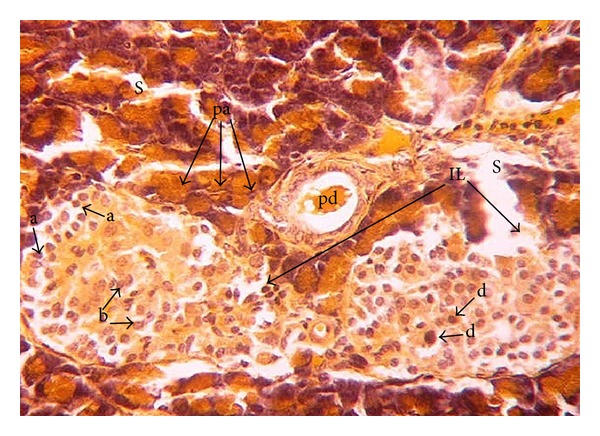
Photomicrograph of pancreas section of normal rat offspring after birth. The parenchyma is divided by septa (s) into pancreatic acini (pa) and pancreatic duct (pd). The endocrine portion of the pancreas consists of the islets of Langerhans (IL). Alpha (a), beta (b), and delta (d) cells are apparent (×400).

The offspring of normal dams showed after birth (Figure 1) normal morphology and distribution of exo- and endocrine portions. The islets of Langerhans are delineated incompletely and separated from the exocrine part by fine reticular fibers. The cells are irregularly distributed within the islets. Three main cells types, alpha, beta, and delta cells, can be distinguished easily. Alpha-cells are small in size, have dark nuclei, and are lying at the periphery of the islet. Beta cells are larger in size showing a faintly stained nucleus. They are more abundant than alpha cells and are found mainly in the center (core) of the islets. Delta cells are characterized by an intermediate cell size and a dark stained nucleus. They are rare in number and are lying near to the alpha cells. One week after birth, control dam offspring showed much larger islets of Langerhans than that after birth. The number of beta-cells has increased, while the number of alpha and delta-cells seemed to remain constant (Figure 3). Two weeks after birth, the islets revealed the normal histology and distribution of alpha, beta, and delta-cells (Figure 5).

**Figure 2:**
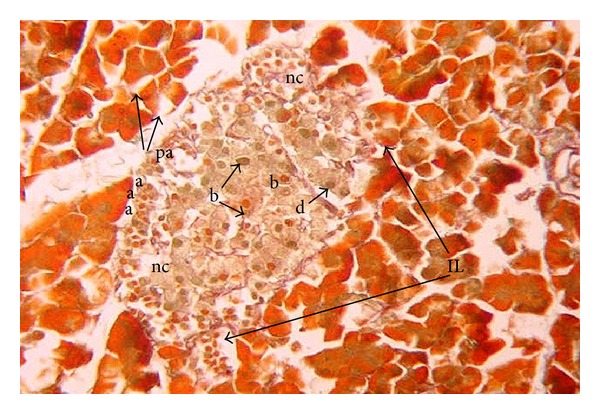
Photomicrograph of pancreas section of diabetic rat offspring just after birth showing disturbed islets of Langerhans (IL) with necrosis (nc) and vacuolations (v). Beta cells (b) are hypertrophied, but low in number. Alpha (a) cells are large in number compared to their corresponding control, (pa) pancreatic acini (×400).

**Figure 3:**
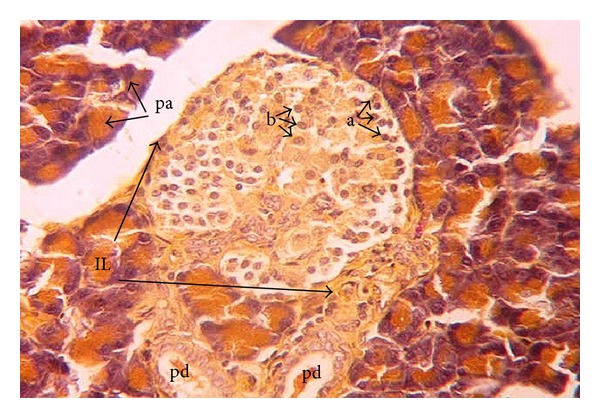
Photomicrograph of pancreas section of normal rat offspring after one week of birth. Islets are intact and larger than after birth with higher numbers of beta (b) cells and fewer numbers of alpha (a) cells. Pd: pancreatic ductile; IL: islets of Langerhans; pa: pancreatic acini (×400).

In offspring of diabetic dam at birth, the islets of Langerhans were hypertrophied and incompletely delineated by reticular fibers. The different cell types showed a disturbed arrangement. A large number of normally sized alpha-cells were found at the periphery of the islet. Beta-cells were hypertrophied. Vacuolation and necrosis were also observed within the endocrine portion of the tissue (Figure 2).

**Figure 4:**
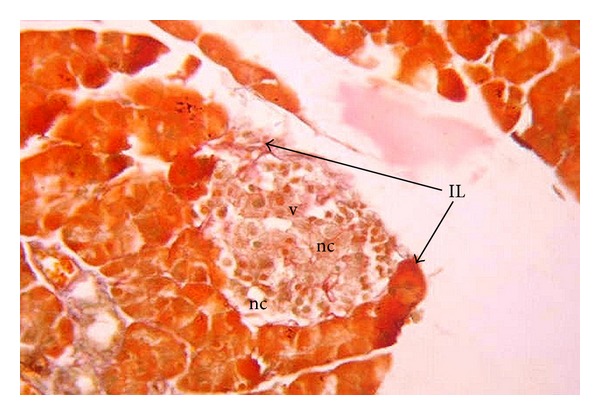
Photomicrograph of pancreas section of diabetic rat offspring after one week of birth showing very small islets of Langerhans (IL) with many necrotic foci (nc) and vacuolations (v) (×400).

**Figure 5:**
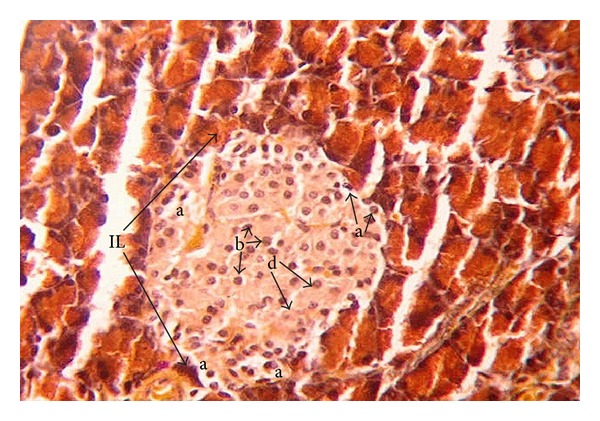
Photomicrograph of pancreas section of normal rat offspring after two weeks of birth illustrating intact islets of Langerhans (IL) with many beta cells (b). Alpha (a) cell, and delta (d) cells are clearly observed (×400).

**Figure 6:**
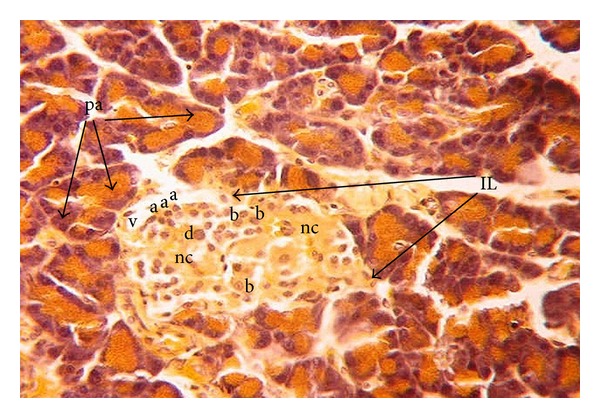
Photomicrograph of pancreas section of diabetic rat offspring after two weeks of birth showing severe degenerative changes. Many necrotic foci (nc) vacuolations (v) are noticed in the islets (IL); the number of beta-cells (b) is reduced and the hypertrophied ones are degenerating. Alpha-cells, delta-cells, and pancreatic acini (pa) are indicated by symbols a, d, and pa, respectively, (×400).

After one week, the islets of Langerhans seemed to be even smaller than at birth (Figure 4). Beta-cells were still hypertrophied. Numerous necrotic foci and vacuolation were observed. After two weeks, the endocrine pancreas of diabetic rat offspring showed severe degenerative changes with increased necrotic foci and vacuolations (Figure 6). Beta cells were reduced in number. Smaller insulin secreting cells predominated, while hypertrophied beta cells were degenerating. Alpha and delta cells were noticed in apparently regular numbers.

#### 3.2.2. Liver

The amount and distribution of carbohydrates granules were estimated in offspring of both groups.

**Figure 7:**
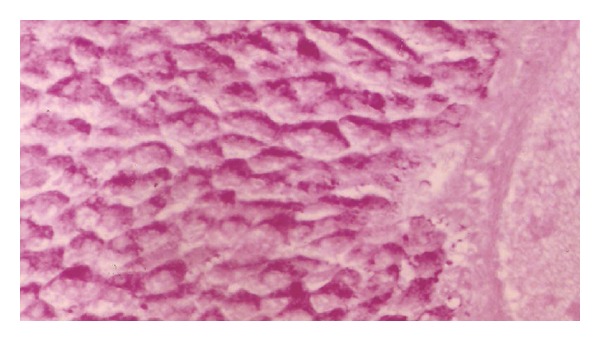
Photomicrograph of liver section of normal rat offspring after birth showing a moderate amount of carbohydrate granules (×400).

Normal rat offspring showed at birth a moderate amount of carbohydrate granules (Figure 7). After one week, the amount was increased (Figure 9). After two weeks, the total carbohydrate amount was much higher than in the weeks before (Figure 11).

**Figure 8:**
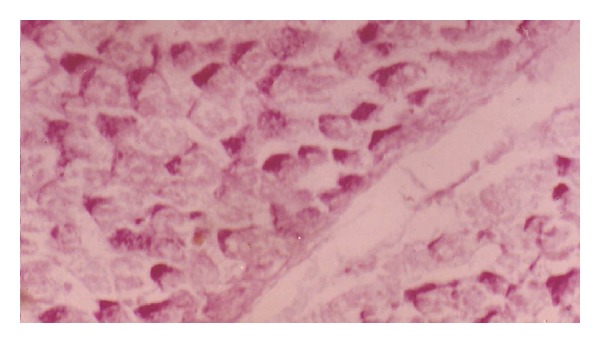
Photomicrograph of liver section of diabetic rat offspring after birth. The stain is less intensive (×400).

**Figure 9:**
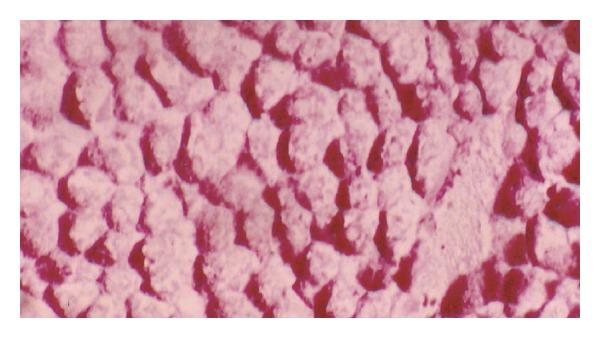
Photomicrograph of liver section of normal rat offspring after one week of birth (×400).

**Figure 10:**
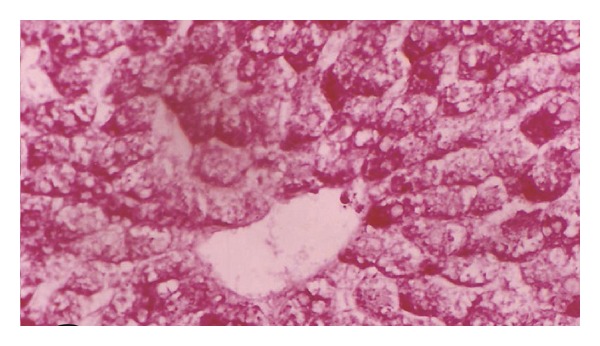
Photomicrograph of liver section of diabetic rat offspring after one week. Notice the difference in distribution and intensity of stain (×400).

**Figure 11:**
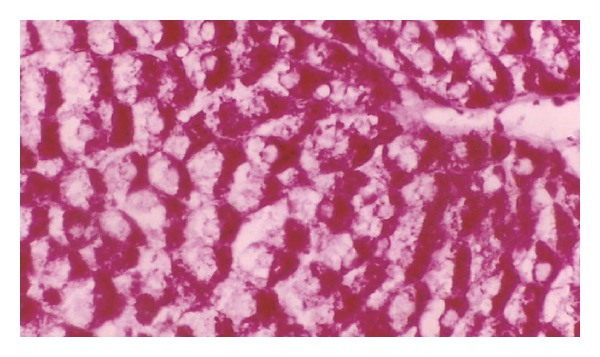
Photomicrograph of liver section of normal rat offspring after two weeks of birth. The stain intensity is much higher than after one week of birth (×400).

**Figure 12:**
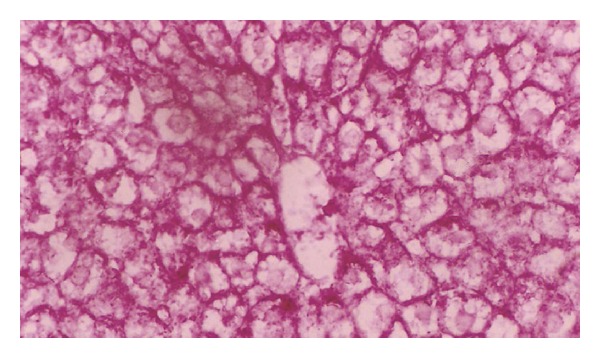
Photomicrograph of liver section of diabetic rat offspring after two weeks of birth. The stain is less intensive than after one week of birth (×400).

Diabetic dam offspring revealed at after birth a much smaller amount of carbohydrate granules compared to control (Figure 8). After one week, the amount of carbohydrate was greater than after birth, but not as high as in control. The granules were more randomly distributed than in the control group (Figure 10). After two weeks, the amount of granules was very small compared to the amount found in liver section of the other time intervals and also much smaller than in normal offspring (Figure 12).

## 4. Discussion

The worldwide increase in the incidence of diabetes in women at reproductive age is the bases for the growing interest in the use of experimental diabetic models in order to gain insight into the mechanisms of induction of developmental alterations in embryos and the effects on newborn. Using an appropriate animal model, several important aspects of human diabetic pregnancies such as the increased rates of spontaneous abortions, malformations, fetoplacental impairments, and offspring diseases in later life can be approached [7, 24]. Therefore, the present study tends to investigate the effect of streptozotocin-induced diabetes before pregnancy and preexisting maternal diabetes on serum glucose, insulin concentration, insulin resistance, insulin sensitivity, and *β*-cell function of offspring at different postnatal periods.

The present study indicated that the pancreatic islet architecture of normal offspring became more organized with the advanced age from the 1st postnatal day to the end of the 2nd week. This is in concurrent with Quinn et al. [25] who examined the histological and ultrastructural differences in the endocrine pancreas in fetal (throughout gestation) and neonatal baboons.

The histological examination of the pancreas in the present study also revealed significant changes in the endocrine tissue of offspring of diabetic dams. At birth, the islets of Langerhans were relatively increased in size and *α*-cells were abundant as compared to the corresponding control offspring. Beta-cells were reduced in number, but not in size; they were actually hypertrophied. Thereafter, beta-cells degenerated and decreased in numbers. Finally, they were replaced by smaller insulin secreting cells. The islets exhibited a profound decrease in their size with many vacuolations and necrosis at 2nd postnatal week.

In accordance with the present study, Aerts et al. [26], Reusens-Billen et al. [27], and Reusens and Remacle [28] reported that at birth, the newborn of diabetic mothers had an enhanced percentage of pancreatic endocrine tissue due to hyperplasia and hypertrophy of the islet cells, leading to a higher beta-cell mass. In our opinion, these alterations may be due to the previous exposure of pancreatic islets to hyperglycemic intrauterine condition before birth. The hypertrophy and hyperplasia of the insulin producing beta-cells have been recognized by many other publications as typical features in fetuses and newborn babies of diabetic mothers [29–31]. As the postnatal age advanced, the beta cells seemed to be almost degranulated leading to low pancreatic insulin content and low plasma insulin [32–34]. Similar endocrine pancreases/beta-cell alterations with low beta-cell mass have been reported in fetuses from spontaneous diabetic GK rats [35, 36].

In contrast to the present results, Rodríguez et al. [37] described that on the first day after parturition, the pancreas section areas and islets' size in pups from mildly and severely diabetic mothers were smaller than those in neonates from nondiabetic controls, and that on day 5 after delivery, the areas of islets of Langerhans in offspring from normal mothers decreased and those in pups from diabetic mothers tended to normalize. Those authors attributed these changes in the islets' size to that after parturition; the offspring is no longer exposed to the high blood sugar levels found in diabetic mothers; thereby, no hyperinsulinemia is needed. As time elapses, then, the area of their Langerhans islets tends to normalize. Furthermore, Han et al. [13] found that islet insulin and glucagon staining and islet features in 15-wk-old STZ-offspring were not significantly different from results shown in the control groups. Thus, these data indicate that maternal diabetes did not affect islet morphological features and insulin synthesis. These findings are in discordance with the present study which indicated a profound decrease of the islet size and islet cells' number of 1 week and 2 weeks postnatal age in offspring of diabetic dams.

Diabetic pregnancy results in several metabolic and hormonal disorders, both in the embryo and the fetus of different species, including humans [38].

The present biochemical investigation of diabetic female offspring revealed a steady and significant increased serum glucose concentration from birth to the end of the experimental period, associated with significantly elevated serum insulin levels at birth and a steadily and significantly decreasing hormone concentration thereafter. Concomitant with these changes in insulin, HOMA beta-cell function was significantly decreased in a time-dependent manner. In addition, the offspring of diabetic mothers revealed a state of tissue insulin resistance and decreased insulin sensitivity. This was indicated by the increase in HOMA-IR and the decrease in HOMA-IS as revealed in this study. HOMA-IR increased, HOMA-IS decreased slightly, and HOMA beta-cell function declined sharply.

These biochemical results are in parallel with the histological observations of the endocrine pancreas. The insulin producing beta-cells were hypertrophied at birth, degraded and reduced in number thereafter, and, finally, replaced by small beta-cells associated with the obtained decline in HOMA beta cell function. The decrease in glycogen content detected in the liver of offspring born from diabetic dams by periodic acid-Schiff (PAS) stain may be secondary to insulin resistance and impaired insulin secretion as indicated in the present study.

The present results are in accordance with observations of several research groups. Insulin secretion has been reported to be abnormal in islets from offspring of diabetic mothers [5, 39], and glucose metabolism was found to be the primary regulator of beta-cell function [40, 41]. Ezenwaka et al. [42] also observed that maternal diabetes alters the morphology, number, and size of offspring islets as well as the ability of the offspring islets to respond to the challenge of pregnancy. Infants of diabetic mothers experience higher levels of glucose during gestation, resulting in pancreatic islet hypertrophy and beta-cell hyperplasia with increased secretion of insulin [42, 43] and proinsulin factors (insulin-like growth factor- (IGF-)1, insulin-like growth factor-binding protein- (IGFBP-)3) [31]. As the age elapsed to one and two weeks, the offspring is no longer exposed to the high blood sugar levels found in diabetic mothers; this may lead to decreased response of beta-cells to glucose stimulus, degranulation of beta-cells, and decreased size of pancreatic islets as noticed in the current study in offspring of diabetic dams at the end of 1 and 2 postnatal weeks.

 Insulin resistance and beta-cell dysfunction are also described in first-degree offspring of type 2 diabetic patients in response to oral glucose challenge. Beta-cell impairment exists in insulin-sensitive offspring of patients with type 2 diabetes, suggesting beta-cell dysfunction to be a major defect determining diabetes development in offspring of diabetic mothers [44]. Long-term effects of intrauterine exposure to diabetes seem to be independent from maternal type of diabetes (T1DM, T2DM, and GDM) as described by Poston [45] and increase risks of obesity [46–50], impaired glucose tolerance [46], and development of NIDDM later in life [51].

Studies on offspring of streptozotocin- (STZ-) induced diabetic rat mothers showed that insulin secretion was significantly impaired in offspring 15 weeks of age. Consistent with these changes, islet glucose metabolism and some important glucose metabolic enzyme activities were reduced; these deteriorations in glucose metabolism may be the cause of impaired beta-cell function in adult STZ offspring [52].

In conduction with the previous publication, many of the animal data on the long-term impact of maternal diabetes come from studies of two types of exposure to hyperglycemia *in utero*: (a) hyperglycemia caused by maternal diabetes that is induced by chemical destruction of pancreatic beta-cells and (b) hyperglycemia produced by a chronic infusion of glucose into mothers during late gestation. All of the studies have been conducted on rats. Three different groups of investigators in addition to the present study have revealed that offspring of rats with overt [7, 53] or mild diabetes [54] induced by streptozotocin administration before conception manifested insulin resistance and reduced insulin sensitivity when studied as adults or later on after birth. Van Assche et al. [8] have reported that the adult offspring are not only insulin resistant but also glucose intolerant. The female offspring developed mild hyperglycemia when they became pregnant, setting the stage for multiple transgenerational transmission of diabetes by nongenetic mechanisms [55]. 

In conclusion, the preexisting diabetes mellitus and hyperglycemia before and during gestation may increase risks of glucose intolerance, insulin resistance, and impaired insulin sensitivity and *β*-cell function in offspring. We invite collaborations to investigate the pathways involved in the molecular mechanisms in which insulin resistance and impaired *β*-cell function are produced in offspring of diabetic dams to suggest targets for therapy and prevention of as well as protection against such perturbations.

## Figures and Tables

**Table 1a:** (a) One-way ANOVA

Groups	Periods		
	0 week	1 week	2 weeks
offspring of normal dams	64.4 ± 7.8^a^ (*n* = 15)	98.3 ± 8.3^b^ (*n* = 18)	95.7 ± 7.3^b^ (*n* = 17)
offspring of diabetic dams	81.8 ± 8.8^a^ (*n* = 19)	121.7 ± 6.2^a^ (*n* = 19)	131.9 ± 3.9^a^ (*n* = 18)
% Difference	+27%	+23.8%	+37.8%
*F*-probability	>0.05	<0.001	<0.001
LSD at 5% level	—	20.96	16.64
LSD at 1% level	—	**28.23**	**22.4**

**Table 1b:** (b) Two-way ANOVA

Effect of time	Effect of diabetes	Time-diabetes interaction
*P* < 0.001	*P* < 0.001	*P* > 0.05

Data are given as mean ± SE; means with the same superscript are not significantly different.

0 week = at birth; 1 week = end of the 1st postnatal week; 2 weeks = end of the 2nd postnatal week; number of observations; % difference: difference between offspring of normal and diabetic dams.

**Table 2a:** (a) One-way ANOVA

Groups	Periods		
	0 week	1 week	2 weeks
offspring of normal dams	1.75 ± 0^d^ (*n* = 6)	2.9 ± 0.001^b^ (*n* = 6)	3.43 ± 0.09^a^ (*n* = 6)
offspring of diabetic dams	2.4 ± 0.06^c^ (*n* = 6)	1.75 ± 0.02^d^ (*n* = 6)	1.55 ± 0.02^e^ (*n* = 6)
% Difference	+37.1%	−39.7%	−54.8%
*F*-probability	<0.001	<0.001	<0.001
LSD at 5% level	0.14	0.14	0.14
LSD at 1% level	**0.19**	**0.19**	**0.19**

**Table 2b:** (b) Two-way ANOVA

Effect of time	Effect of diabetes	Time-diabetes interaction
*P* < 0.001	*P* < 0.001	*P* < 0.001

Data are given as mean ± SE; means with the same superscript are not significantly different.

0 week = at birth; 1 week = end of the 1st postnatal week; 2 weeks = end of the 2nd postnatal week; number of observations; % difference: difference between offspring of normal and diabetic dams.

**Table 3a:** (a) One-way ANOVA

Groups	Periods		
	0 week	1 week	2 weeks
offspring of normal dams	0.29 ± 0.02^b^ (*n* = 6)	0.48 ± 0.02^b^ (*n* = 6)	0.54 ± 0.05^a^ (*n* = 6)
offspring of diabetic dams	0.76 ± 0.06^a^ (*n* = 6)	0.63 ± 0.02^a^ (*n* = 6)	0.60 ± 0.01^a^ (*n* = 6)
% Difference	+162%	+36.9%	+11.1%
*F*-probability	<0.001	<0.001	>0.05
LSD at 5% level	0.14	0.08	—
LSD at 1% level	**0.20**	**0.11**	—

**Table 3b:** (b) Two-way ANOVA

Effect of time	Effect of diabetes	Time-diabetes interaction
*P* > 0.05	*P* < 0.001	*P* < 0.001

Data are given as mean ± SE; means with the same superscript are not significantly different.

0 week = at birth; 1 week = end of the 1st postnatal week; 2 weeks = end of the 2nd postnatal week; number of observations; % difference: difference between offspring of normal and diabetic dams; HOMA-IR: homeostasis model assessment for insulin resistance.

**Table 4a:** (a) One-way ANOVA

Groups	Periods		
	0 week	1 week	2 weeks
offspring of normal dams	151.3 ± 11.8^a^ (*n* = 6)	54.2 ± 3.1^a^ (*n* = 6)	47.2 ± 4.3^a^ (*n* = 6)
offspring of diabetic dams	47.1 ± 1.9^b^ (*n* = 6)	42 ± 0.5^b^ (*n* = 6)	41.1 ± 0.8^a^ (*n* = 6)
% Difference	−68.9%	−22.5%	−12.9%
*F*-probability	<0.001	<0.01	>0.05
LSD at 5% level	26.8	7.03	—
LSD at 1% level	**38.1**	**10**	—

**Table 4b:** (b) Two-way ANOVA

Effect of time	Effect of diabetes	Time-diabetes interaction
*P* < 0.001	*P* < 0.001	*P* < 0.001

Data are given as mean ± SE; means with the same superscript are not significantly different.

0 week = at birth; 1 week = end of the 1st postnatal week; 2 weeks = end of the 2nd postnatal week; number of observations; % difference: difference offspring of normal and diabetic dams; HOMA-IS: homeostasis model assessment for insulin sensitivity.

**Table 5a:** (a) One-way ANOVA

Groups	Periods		
	0 week	1 week	2 weeks
Normal offspring	1.03 ± 0.08^a^ (*n* = 6)	0.97 ± 0.06^a^ (*n* = 6)	1.18 ± 0.1^a^ (*n* = 6)
Diabetic offspring	0.56 ± 0.02^b^ (*n* = 6)	0.24 ± 0.01^b^ (*n* = 6)	0.22 ± 0.004^b^ (*n* = 6)
% Difference	−45.6%	−75.3%	−81.4%
*F*-probability	<0.001	<0.001	<0.001
LSD at 5% level	0.21	0.14	0.25
LSD at 1% level	**0.29**	**0.19**	**0.36**

**Table 5b:** (b) Two-way ANOVA

Effect of time	Effect of diabetes	Time-diabetes interaction
*P* < 0.05	*P* < 0.001	*P* < 0.01

Data are given as mean ± SE; means with the same superscript are not significantly different.

0 week = at birth; 1 week = end of the 1st postnatal week; 2 weeks = end of the 2nd postnatal week; number of observations; % difference: difference between offspring of normal and diabetic dams; HOMA-beta-cell function: homeostasis model assessment for *β*-cell function.
