# Correction to “Maternal Rat Diabetes Mellitus Deleteriously Affects Insulin Sensitivity and Beta-Cell Function in the Offspring”

**DOI:** 10.1155/jdr/9876342

**Published:** 2025-12-02

A-B. M. Aref, O.M. Ahmed, L.A. Ali, M. Semmler, “Maternal Rat Diabetes Mellitus Deleteriously Affects Insulin Sensitivity and Beta-Cell Function in the Offspring,” *Journal of Diabetes Research*, 2013, 429154, https://doi.org/10.1155/2013/429154

In the article, there is an error in Figure 3, which has been inadvertently duplicated from Figure 1d of one of the author's other publications, which was published concurrently [1]. The correct Figure 3 is shown below:

This error does not affect the results or conclusions of the article and has been approved by the editorial board.

We apologize for this error.

## Figures and Tables

**Figure 1:**
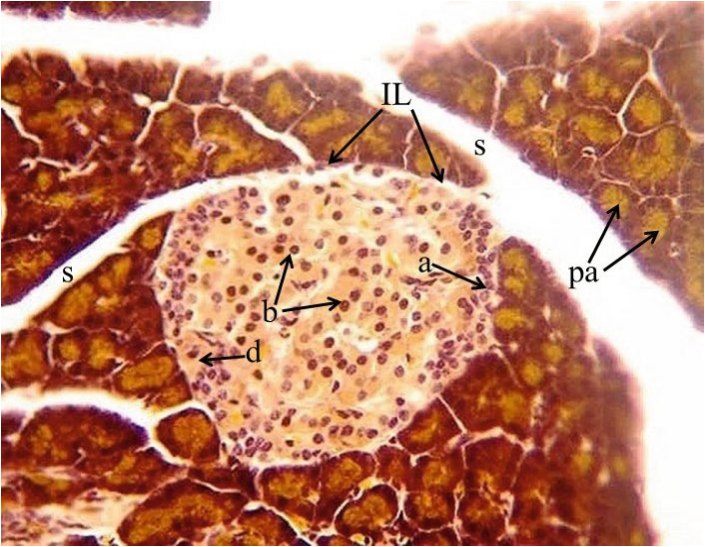
Photomicrograph of the pancreas section of normal rat offspring after 1 week of birth. Islets are intact and larger than after birth with higher numbers of beta (b) cells and fewer numbers of alpha (a) cells. Pd: pancreatic ductile; IL: islets of Langerhans; pa: pancreatic acini (×400).
